# Identification of Plant Peptides as Novel Inhibitors of Orthohepevirus A (HEV) Capsid Protein by Virtual Screening

**DOI:** 10.3390/molecules28062675

**Published:** 2023-03-16

**Authors:** Ghulam Mustafa, Hafiza Salaha Mahrosh, Syed Awais Attique, Rawaba Arif, Mohammad Abul Farah, Khalid Mashay Al-Anazi, Sajad Ali

**Affiliations:** 1 Department of Biochemistry, Government College University Faisalabad, Faisalabad 38000, Pakistan; 2 Department of Biochemistry, University of Agriculture Faisalabad, Faisalabad 38000, Pakistan; 3 School of Interdisciplinary Engineering & Science (SINES), National University of Sciences & Technology (NUST), Islamabad 44000, Pakistan; 4 Agency for Science, Technology and Research (A*STAR), Bioinformatics Institute, 30 Biopolis Street, Matrix, Singapore 138671, Singapore; 5 Department of Biochemistry, University of Jhang, Jhang 35200, Pakistan; 6 Department of Zoology, College of Science, King Saud University, P.O. Box 2455, Riyadh 11451, Saudi Arabia; 7 Department of Biotechnology, Yeungnam University, Gyeongsan 38541, Republic of Korea

**Keywords:** capsid protein, EDGE, MD simulation, molecular docking, ORF2, TDGH

## Abstract

Hepatitis E virus (HEV) is the notable causative agent of acute and chronic hepatic, renal, pancreatic, neurological, and hematopoietic blood cell infections with high risk in immunocompromised patients. Hepatic failure is mostly documented among adults, pregnant women, and patients with preexisting liver disease. HEV is a positive sense RNA virus of 7.2 kb genome size with typically three open reading frames (ORFs) which play essential roles in viral replication, genome assembly, and transcription. The mutational substitution in the viral RNA genome makes more it difficult to understand the actual relationship in the host–virus association. ORFs of HEV encode different structural and non-structural proteins and one of them is the capsid protein which is coded by ORF2. The capsid protein mediates the encapsulation of the viral genome as well as being involved in virion assembly. In the current study, the ligand-based docking approach was employed to inhibit the active amino acids of the viral capsid protein. Depending upon S-score, ADMET profiling, and drug scanning, the top ten tetrapeptides were selected as potential drug candidates with no toxicity counter to HEV receptor protein. The S-score or docking score is a mathematical function which predicts the binding affinities of docked complexes. The binding affinity of the predicted drug–target complexes helps in the selectivity of the desired compound as a potential drug. The best two selected peptides (i.e., TDGH with S-score of −8.5 and EGDE with S-score of −8.0) interacted with the active site amino acids of the capsid protein (i.e., Arg399, Gln420, and Asp444). The molecular dynamics simulations of RMSD trajectories of TDGH–capsid protein and EDGE–capsid protein have revealed that both docked complexes were structurally stable. The study revealed that these tetrapeptides would serve as strong potential inhibitors and a starting point for the development of new drug molecules against the HEV capsid protein. In future, in vivo studies are needed to explore selected peptides as potential drug candidates.

## 1. Introduction

Hepatitis E virus (HEV) or Orthohepevirus A is the causative agent of acute or chronic viral hepatitis infections. The hepatitis E virus belongs to the genus *Orthohepevirus* that further consists of four species, i.e., *Orthohepevirus A*, *B*, *C*, and *D*. Among all four genera, *Orthohepevirus A* is mainly associated with human infections [1]. HEV is responsible for hepatic, neurological, and renal manifestations [2]. It is estimated that about one-third of the world population is at risk of HEV infection with 3.3 million reported cases annually. According to a World Health Organization (WHO) survey, HEV infections caused approximately 44,000 mortalities in 2015. Due to poor prognostication and health conditions, pregnant females are at a higher risk of viral infections with a fatality rate of more than 30%. Moreover, the direct transfer of virus from the infected mother to the fetus causes premature birth [3]. Different zoonotic and oral routes, blood transfusions, organ transplants, and use of infected uncooked raw food have been recognized as modes of transmission of HEV infection with high frequency [4].

The HEV belongs to *Hepeviridae* family which is divided into two genera (i.e., *Orthohepevirus* and *Piscihepevirus*). Over the past few years, many new strains of HEV have been identified, which are not only limited to humans but have also been found in animal species [5]. HEV is a positive and single-stranded RNA virus with a (5′-UTR) region capped at the 5′-end, short 3′-UTR terminated by poly(A) stretch, and typically three open reading frames (ORFs) [6]. The open reading frame 1 (ORF1) encodes a non-structural protein of 1693 amino acids, which is comprised of seven domains including MeT, PCP (papain-like cysteine protease), HVR region, Macro, Y, helicase, and RdRp domains [7,8]. The ORF2 encodes a capsid protein of 660 amino acids, which plays a crucial role in virion assembly, encapsulation of the viral genome, and mediates the attachment to the host cell [9]. ORF3 is involved in the morphological events of the viral cells during the viral exodus [10].

Genome-wise comparative analysis has revealed the extensive genomic diversity among the four major genotypes and other subtypes of HEV [11]. The high variability and frequent mutations in the viral genome have been directly associated with the emergence of most of the HEV infections. Although, all ORF regions of HEV genome are equally important in viral assembly and functioning, ORF2 encodes the essential encapsulating capsid protein of HEV, which protects the viral RNA genome and mediates the adherence to the host cell. Various studies have explored the role of ORF2 in virion maturation and genome assembly. The cellular and structural patterns of HEV capsid protein makes it a compatible target to study host–viral interactions [11,12].

The capsid protein was selected because of its importance in serving as an entry point of virus into the host cell. The capsid protein serves as a major reservoir point for attachment in the host cells and is essential for the assembly of the viral genome. Moreover, this protein enhances the survival and stability of the virus in acidic and alkaline medium [13,14]. Different sequence and structural analyses have reported conserved binding motifs and β-barrel fold structures which contain polysaccharide binding sites among all HEV genomes which are involved in capsid disassembly and binding to host receptors [15]. This makes the HEV capsid protein a potential target to block viral entry into the host cells at an early stage of infection. Therefore, instead of using any phytochemical or synthetic vaccine, we used plant derived tetrapeptides in this study, which are most conserved in reported antimicrobial plant proteins as potent inhibitors of HEV capsid protein.

Ribavirin therapy is highly recommended in HEV infections with a median of 3 months from diagnosis. Different clinical cases reported the failure of ribavirin as the virus is still positive in blood samples of the treated patients and after the first course, the infection again relapsed [16,17]. Anemia was the most common observed side effect in first-line ribavirin therapy. Pegylated interferon-α has also been used successfully for HEV treatment but is associated with many adverse effects including cholestasis and teratogenicity [18,19].

With an increase in resistance events in a variety of microbes because of mutational substitutions and adaptive behavior in the microbial genomes, scientists have shifted their direction of research towards medicinal plants. Plants are the sole source of different peptides, phytochemicals, and small organic molecules with great efficacy and less toxicity [20,21].

In the literature, different peptides have been reported with their potential to bind and inhibit HEV receptor proteins, but the source of these peptides was animals or microorganisms. Peptides from animals such as porcine β-defensin-2 cannot be used in Muslim countries because of religious perspectives. Therefore, in the light of previous studies, we used plants as a peptide source to target the viral capsid protein. Moreover, despite using different nanodrug carriers and developed epitope vaccines for HEV treatment, there is still no assurance regarding patients’ safety. Interferons and ribavirin have been administrated in many clinical cases, but in most of the cases the infection relapsed, or the patients did not respond to the drugs that ultimately led to the failure of these therapies. If the infection is overcome at an early stage by targeting the viral entry into the host cell, the survival rate could be extended. The aim behind the current study was to target the capsid protein in order to inhibit viral–host receptor interactions.

In the current study, 100 tetrapeptides devised from 500 antimicrobial peptides were docked to the capsid protein of HEV. The online database NCBI’s Entrez Protein was used to access the protein sequences of plant-derived antimicrobial peptides. In order to find the most conserved protein sequences among all 500 antimicrobial peptides, multiple sequence alignment (MSA) was performed via the MEGA tool. The prediction of conserved sequences of antimicrobial peptides from plants of different families, narrowed down the selection of ligands from the pool of reported antimicrobial peptides. MSA highlights the most repeated sequences which could be used as potential antimicrobial tetrapeptides to counter different viral, bacterial, and fungal infections. The aim of this study was to reveal the antimicrobial nature of screened tetrapeptides against the capsid protein of HEV by molecular docking and a molecular dynamics simulation approach. On the basis of docking scores and drug assessments, top hits were selected as potential drug candidates against the capsid protein of HEV.

## 2. Results

### 2.1. Molecular Docking and Interaction Analysis

**Figure 1:**
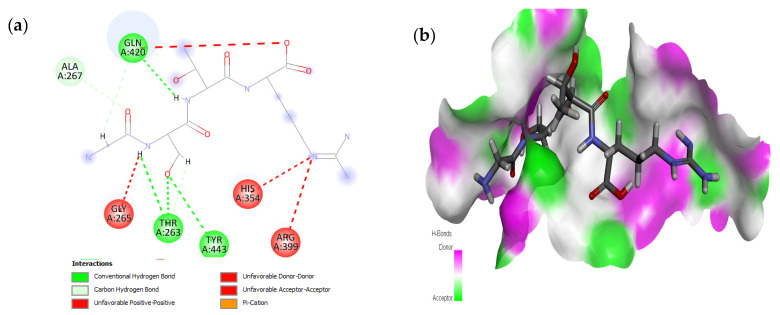
Interaction (**a**) and binding pattern (**b**) of GSTR peptide with ORF2 (capsid protein) with HEV as receptor.

The active site residues (i.e., Arg366, Arg399, Gln420, Gln421, Asp444, and Gln446) were selected on the basis of previously reported studies [22]. A total 100 antimicrobial tetrapeptides were docked to the receptor protein (capsid protein) of HEV. The binding pattern and S-scores of protein–ligand docking screened out the top ten hits as potent inhibitors of the capsid protein of HEV by integrating with active site residues. Capsid protein protects the virion and mediates the attachment to the host cell. Therefore, capsid protein could be the most appropriate target to hinder HEV infection [4]. All the best selected peptides in this study exhibited strong interactions with active site residues of the catalytic site of the capsid protein as a receptor (Table 1). In the current study, the peptide GSTR with S-score of −10.1 showed interactions with Thr263, Gln420, and Tyr443 residues of the binding pocket (Figure 1).

**Figure 2:**
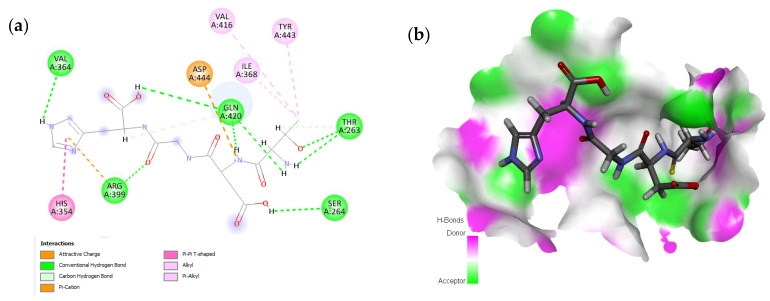
Interaction (**a**) and binding pattern (**b**) of TDGH peptide with ORF2 (capsid protein) with HEV as receptor.

**Figure 3:**
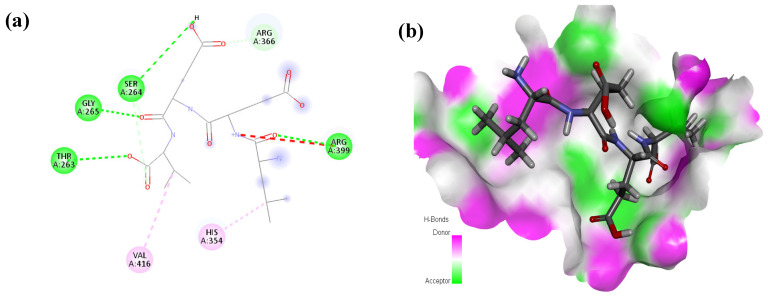
Interaction (**a**) and binding pattern (**b**) of LEEV peptide with ORF2 (capsid protein) with HEV as receptor.

The peptide TDGH established conventional hydrogen bonds with amino acids Thr263, Ser264, Val364, Arg399, and Gln420 of the binding pocket of the capsid protein. The amino acids Ile368, Val416, and Tyr443 were involved in alkyl and pi-alkyl interactions and the amino acid His354 was involved in Pi-Pi T-shaped interaction with the receptor protein (Figure 2). Similarly, the amino acids Thr263, Ser264, Gly265, and Arg399 of the receptor protein were involved in conventional hydrogen bonds with the peptide LEEV (Figure 3). The interactions and binding patterns of the other seven peptides (i.e., WDDG, EGDE, FTDG, EPST, SDAF, PRGS, and NTFP with the ORF2 (capsid protein) of HEV are shown in Figures S1–S7 of the Supplementary Materials.

### 2.2. Drug Scanning and ADMET Profiling

The Lipinski’s rule of five (Ro5) illustrates the parameters to distinguish drug-like and nondrug-like behavior of chemical compounds. In order to assure drug-like behavior, a drug must have molecular mass (≤500 Dalton), hydrogen bond donor (<5), hydrogen bond acceptor (<10), molar refractive index (40–130), and log P (≤5). All selected top ten tetrapeptides fully accomplished the drug-like criteria and violated one rule (Table 2). A compound could be considered a potential drug candidate, if it violates no or one rule out of five [23]. To further assess the pharmacological potential of these ten tetrapeptides, the admetSAR tool was used to predict the ADMET based attributes of these peptides from a medical perspective. The ADMET based evaluation confirmed the bioavailability and lack of toxicity of these peptides (Table 3). Based on the overall profiling, the best selected antimicrobial tetrapeptides could therefore be used as potential drug candidates counter to the capsid protein of HEV.

### 2.3. Molecular Dynamics Simulation

**Figure 4:**
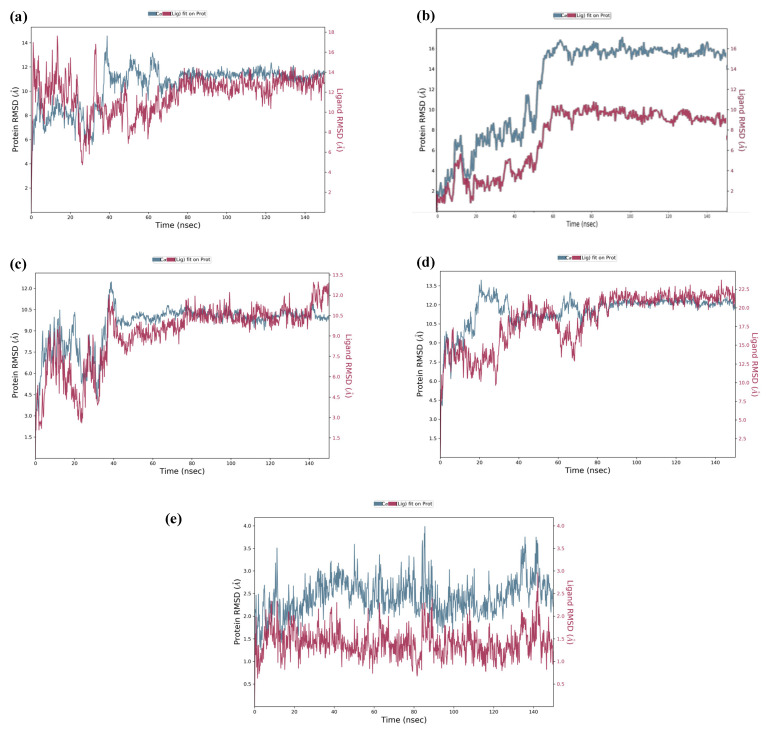
Molecular simulation RMSD trajectories with respect to time. The MD simulation was done for 150 ns at 310 K temperature, 1.00314 bar pressure, 1000 frames, and 7.4 pH). The MD simulation of the docked complex of GSTR–capsid protein (**a**); TDGH–capsid protein (**b**); LEEV–capsid protein (**c**); WDDG–capsid protein (**d**); EGDE–capsid protein (**e**). The left Y-axis shows the variation of protein RMSD through time. The right Y-axis shows the variation of ligand RMSD through time.

The top five hits from the molecular docking study were selected for the molecular dynamics (MD) simulation study for the determination of binding characteristics of these peptides as ligands in the protein binding pocket in the dynamic state. The molecular dynamics simulation was performed using Schrodinger’s Desmond Module to check the stability of peptide–protein docked complexes. In theMD simulation of protein–GSRT peptide complex, both protein and the ligand showed fluctuations up to 65 ns but after 65 nm the ligand was bound firmly in the protein binding pocket (Figure 4a).

For the protein–TDGH peptide complex, based on RMSD plots, the protein was found to be stabilized at around 16 Å after a considerable fluctuation from 8 to 16 Å, which showed that the protein had undergone conformational changes during the simulation (Figure 4b). However, the protein was stabilized towards the end of the simulation which revealed that the protein overall was stable. The RMSD of the ligand indicated that it followed the conformational changes of the backbone from the protein, which gives information about where the protein is showing its binding activity within its binding site. The peptide showed fluctuations up to 62 nm and after that the MD simulation elucidated that the peptide TDGH has made a stable complex with the active residues of the binding pocket of the capsid protein. The simulation trajectories of TDGH and the receptor protein did not show any fluctuation after 62 ns of the simulation time frame. A change of only 1 Å (i.e., from 16 Å to 15 Å) was observed during the simulation. The change of 4 Å in RMSD value is universally assumed as acceptable for the docked complex under given temperature, pressure, and salt conditions. Since the fluctuation of the ligand was not found to be higher than that of the protein, it could be concluded that the ligand was stabilized within the binding pocket of the protein without overlap.

The MD simulation of the protein–LEEV peptide complex revealed that the ligand was firmly bound to the protein throughout the simulation time of 150 ns (Figure 4c). Initially, fluctuations were observed in both protein and the ligand, but after 42 ns no significant RMSD fluctuations were revealed. In the case of the protein–WDDG complex, the MD simulation showed that both protein and ligand exhibited fluctuations up to 38 ns but after that the complex became stabilized for the simulation time (Figure 4d). Another fluctuation was observed at 61 to 70 ns which was not significant. The ligand stayed firmly bound to the protein binding pocket.

Similarly, the MD simulation RMSD trajectory of EGDE–capsid protein complex revealed that the docked complex is structurally stable with RMSD values of 1.5 Å to 3.5 Å (i.e., the change in RMSD is below 3 Å) throughout the 150 ns time period (Figure 4e). The combined RMSD of the protein–ligand complex during MD simulation also remained stable throughout the simulation time frame of 150 ns as the combined trajectories did not show any notable fluctuations (only shown at a short time frame from 82 ns to 86 ns) in the trajectory or RMSD change. From this stability of the complex, it is elucidated that with respect to time, the docked complex is structurally stable after making hydrogen bonds and other interactions.

**Figure 5:**
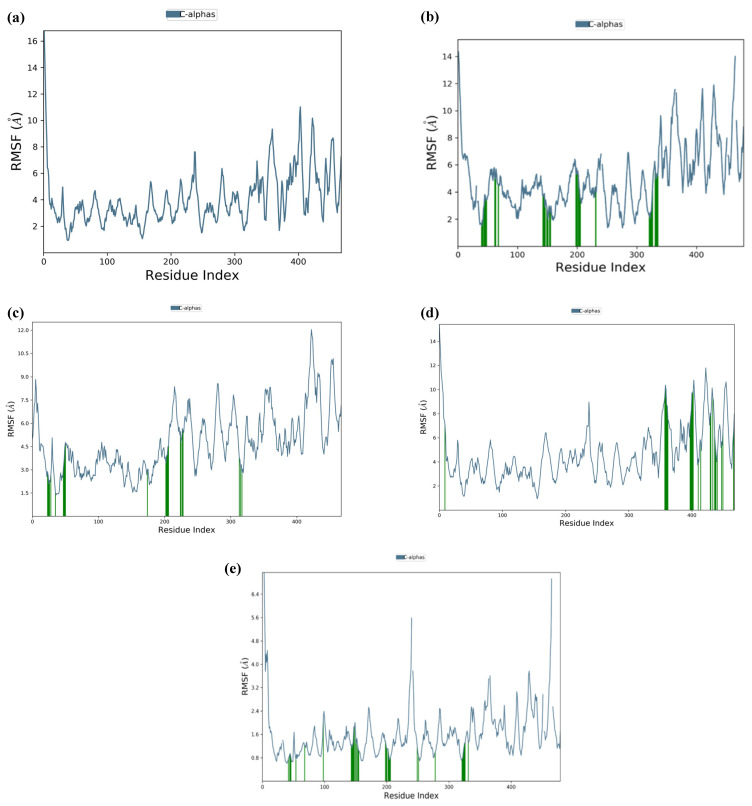
Residue wise Root Mean Square Fluctuation (RMSF) of protein–ligand complexes during 150 ns. (**a**) capsid protein with GSTR peptide; (**b**) capsid protein with TDGH peptide; (**c**) capsid protein with LEEV peptide; (**d**) capsid protein with WDDG peptide; (**e**) capsid protein with EGDE peptide. The RMSF of docked protein showed no fluctuation of interacting residues, where green vertical lines are representing interacting residues.

The consistency or H-bond interaction stability of key residues of the receptor protein with the top five peptides throughout MD simulation showed that all the peptides in complex with capsid protein are stable and could be used as potential hits for drug design against HEV (Figures S8–S12). The Root Mean Square Fluctuation (RMSF) of the top five peptides in complex with capsid protein revealed stable peaks throughout the 150 ns MD simulation of key interacting residues with the peptides (Figure 5). The findings are strong evidence supporting the stability of all peptide–capsid protein docking complexes.

Some precocious fluctuations which were observed in RMSD and RMSF trajectories were not found to be drastic in most of the cases. The fluctuations might reflect some necessary conformational modifications in the enzyme complex which can serve in an appropriate redirection and reposition of each peptide as ligand inside the catalytic binding pocket or cavity. These modifications normally take some nanotime (ns) to make very strong and stable molecular interactions and noncovalent bonds.

**Figure 6:**
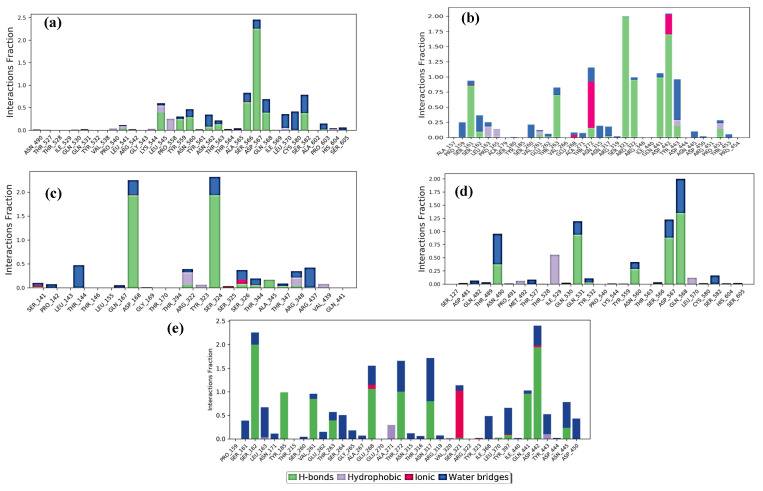
Protein–ligand contact histogram. Protein structures complexed with (**a**) GSTR peptide; (**b**) TDGH peptide; (**c**) LEEV peptide; (**d**) WDDG peptide; (**e**) EGDE peptide.

The Protein Ligand Interaction Fingerprints (PLIF) of the peptide GSTR and capsid protein complex before and after MD simulation revealed that the amino acid Asp567 is important to stabilize the complex for drug design as it is involved in making an H-bond (Figure 6a). The peptide TDGH and capsid protein complex before and after MD simulation showed that Ser321 and Arg322 are also important in the stability of the complex for drug design, while GLN441 and Asp442 are making consistent H-bonds before and after MD simulation (Figure 6b). The PLIF interaction diagram of LEEV–capsid protein complex before and after MD simulation showed that Asp168 and Ser324 are important residues in the stability of the complex as these amino acids are making H-bonds with the peptide (Figure 6c). In the protein–WDDG peptide complex, Gln531, Asp567, and Gln568 were revealed to be important in making H-bonds with the peptide WDDG and stabilizing the complex (Figure 6d).

Similarly, the EGDE–capsid protein complex before and after MD simulation showed that the residues Ser162, Tyr185, Glu268, Thr272, Ser321, Gln441, and Asp442 are important residues in the stability of the complex for drug design. The residues Ser162, Tyr185, Glu268, Thr272, Gln441, and Asp442 are making H-bonds with peptide EGDE as well as showing interactions with water molecules of a defined solvent periodic boundary. The amino acids Ser162 and Asp442 have tentatively contributed to making interactions with the EGDE peptide (Figure 6e).

## 3. Discussion

The molecular docking approach has been used to reveal the binding potential of different candidates as ligand molecules to a variety of receptor proteins. Different kinds of docking approaches have been practiced according to the type and kind of analysis and receptor–ligand type. The docking studies execute the interactions, energy validations, and patterns of binding of ligands to the catalytic site of receptor moieties. Computer assisted drug discovery can assist the trial of different libraries of ligand molecules against different proteins as targets [24]. The maximum occupancy of the binding pocket, strong H-bonds, and minimum energy structure confirm the potential of ligand molecules with active residues of the receptor protein. Computational studies help scientists to estimate the results of the proposed study before starting experimental studies [21].

HEV infection can lead to chronic conditions with severe hepatic and renal complications. Until now, four genotypes of HEV have been recorded, mainly responsible for most of the infection cases. HEV genotypes 1 and 2 are obligate human pathogens, while types 3 and 4 have zoonotic origins. Although, acute infection is self-limiting, the chronic condition is the most dangerous and surprisingly an increased number of cases have made it a leading cause of high mortality worldwide [25]. The patterns of human HEV infections have two distinctive pathways. HEV genotypes 1 and 2 are transmitted via blood transfusions, poor sanitations, fecal-oral routes, and most prominently via contaminated water [26]. The other two genotypes (i.e., 3 and 4) mainly have zoonotic modes of transmission [27]. The open reading frames (ORFs) of the viral genomes hold a great importance in RNA genome assembly and encoding structural and non-structural proteins. ORF2 encodes the capsid protein which is involved in the encapsulation of the viral genome. The inhibition of capsid protein hinders the genome assembly and structural domain of HEV.

In the current study, ORF2 which encodes the capsid protein, was targeted to inhibit viral attachment to the host. A sequence identity of 80–85% of capsid proteins with all four main genotypes makes HEV-ORF2 a good target for vaccine development. Unfortunately, until now, many preclinical trials have failed due to poor clinical management and insufficient or mixed data about the HEV RNA genome. The lack of knowledge about proteolytic processing and HEV capsid protein generation acts as a major obstacle in the development of a potent drug or vaccine [15]. Different studies have demonstrated the relation of HEV-ORF2 in different interspecies infection cases. Nguyen et al. [28] reported the partial capsid protein region of 456–605 amino acids encapsulated with the P-domain from the chimeric HEV-3 strain. Despite knowing the mechanism, the relationship between viral capsid proteins and cellular receptor determinants are still underlying and need further investigation.

A study has revealed two different protein products of ORF2 in HEV3 Kernow-C1 p6 strain-infected HepG2/C3A cells. The ORF2s (secreted protein ORF2) was the first secreted form of ORF2, which was fully translated from start to stop codon as a full-length protein, while the second form was the capsid-associated truncated one which was partially translated as initiated from an internal starting codon (Yin et al., 2018). Furthermore, another study on the Kernow-C1 p6 strain has also demonstrated three isolated forms of ORF2 (i.e., two glycosylated forms related to a secretory pathway and one non-glycosylated form during the viral replication cycle). Although, the discovery of these forms of ORF2 are essential to understand the viral mode of action, the biological functions, catalytic and cleavage sites are still unknown [29]. In short, these reported changes have indicated the advanced protease processing and posttranslational modifications in the viral machinery which generate different ORF forms of HEV [15].

In this study, the top ten selected tetrapeptides (i.e., GSTR, TDGH, LEEV, WDDG, EGDE, FTDG, EPST, SDAF, PRGS, and NTFP) showed one violation each when checked for their drug-like properties according to Lipinski’s Ro5. Similarly, all tetrapeptides also fulfilled their ADMET related drug-like parameters for being good drug candidates. Recently, a variety of peptides have been used as potential inhibitors of different viral and bacterial proteins [30]. Plant-derived peptides and secondary metabolites have been reported with a variety of functional properties as potent drug candidates against a variety of microbial receptor proteins [20]. Quintero-Gil et al. [14] predicted six antiviral peptides of length 12 to 15 amino acids from nisin, porcine β-defensin 2, and subtilosin sequences and docked to the capsid protein of HEV. Using different computer-aided drug discovery (CADD) approaches, these predicted antiviral peptides displayed strong interactions with the active residues of the binding pocket of HEV capsid protein with low toxicity based on ADMET profiling. Similarly, Glitscher et al. [31] studied the potential inhibitory impact of silvestrol on infectious particles of hepatitis E virus. The HEV-infected A549 cell lines were treated with silvestrol and the released number of HEV genomes were observed by real-time PCR. Different concentrations of silvestrol were subjected to variable time intervals to check the reduction in viral RNA load. The outcomes of the study demonstrated the inhibitory effect of silvestrol with notable reduction in HEV protein synthesis and release of viral particles.

In another study, Galani et al. [22] investigated eight licensed antimalarial drugs and two anti-hepatitis C virus agents (i.e., sofosbuvir and ribavirin) as inhibitors of five different target proteins of the HEV genome. These ten compounds were docked counter to RdRp, zinc-binding non-structural protein, capsid protein, cryoEM structure of HEV VLP, and the E2s domain of HEV genome. The binding patterns of all tested compounds were found to be different to each other on the basis of their docking scores but among all, N-desethylamodiaquine, amodiaquine, and lumefantrine showed great binding affinity with different proteins of HEV. Similarly, Das et al. [32] evaluated the binding patterns of ten plant-derived active phytochemicals found in different medicinal plants. The compounds (i.e., glycyrrhizin, vasicinone, lignans, galactomannan, zingerone, piperine, wedelolactone, cajanin, catechin, and gallic acid) were docked to the 32 core proteins of the HEV genome. The results of the study indicated the hepatoprotective nature of these active compounds with no cytotoxic or adverse effects [32]. Zhang et al. [33] targeted the HEV-3 RNA via vector-based multiplexed shRNAs to thwart the viral genome from adapting with new mutation(s). The shRNAs directly inhibit the methyltransferase domain of the ORF2 region to hinder the viral encapsulation, replication, and translation processes. The combinational therapy of RNA interference and AAV vector-based gene therapy has great potential to suppress HEV replication.

Taken together, all literature studies show there is still a dire need of such a mechanistic approach to fully understand the underlying biological and functional processes of HEV genomic machinery. In the current study, the capsid protein of HEV was selected as a target receptor protein because of its variable nature and involvement in viral encapsulation. Capsid protein serves as an entry point to attach to host receptor cells and is also essential for the assembly of viral particles. Because of this function, the HEV capsid protein was selected to block the virus–host receptor interactions.

For this study, the sequences of 500 antimicrobial peptides were retrieved from the protein database of NCBI using the filter of plant species. The FASTA format of all peptide sequences were imported to MEGA software for multiple sequence alignment to determine the most conserved tetrapeptides from the literature citing antimicrobial peptides. In total, 100 tetrapeptides were devised and docked to the catalytic pocket of the capsid protein. On the basis of S-scores and binding patterns of ligand–receptor complexes, the top ten tetrapeptides were selected and assessed for bioavailability. Among all hits, the peptide GSTR with a S-score of −10.1 showed strong interactions with Thr263, Gln420, and Tyr443 residues of the catalytic pocket. Similarly, the peptides TDGH, LEEV, and WDDG with S-scores of −8.5, −8.4, and −8.2 showed interactions with (Thr263, Ser264, Val364, Arg399, and Gln420), (Thr263, Ser264, Gly265, and Arg399), and (Arg366, Arg399, and Gln420) residues of the binding pocket of the receptor protein (i.e., capsid protein). The active site amino acids Arg399 and Gln420 were found in the interactions of both TDGH and EGDE peptides with the capsid protein. Following the same pattern of interactions, the remaining tetrapeptides also showed maximum occupancy of the binding pocket. The druggability and ADMET profiling of the best selected peptides showed satisfactory results with no toxicity. All tetrapeptides followed Lipinski’s rule of five with one violation only. The fluctuations in one rule can be acceptable as the ADMET assessment of selected compounds showed no Ames toxicity and carcinogenicity. The molecular dynamics simulation study also confirmed the stability of TDGH and EGDE peptides with capsid protein as a ligand–protein complex. Overall, these peptides could be used as potential drug candidates to counter the capsid protein of HEV.

The tetrapeptides which have been identified in the current study via computational approaches will provide a basis for the development of novel treatment options against HEV and therefore, can be assessed experimentally. Taken together, the tetrapeptides (i.e., GSTR, TDGH, LEEV, WDDG, EGDE, FTDG, EPST, SDAF, PRGS, and NTFP) exhibited good results in the current in silico study and could be further analyzed as potential drug candidates in biological in vitro studies against HEV. Further scientific confirmation after preclinical and clinical research would be a necessary first step to achieve the basic binding profiles of the investigated tetrapeptides, because these peptides exhibited a central scaffold, which could be employed to start a drug designing campaign for the optimization of these natural compounds. It has been revealed in the study that these peptides have no or low toxicity with more capabilities to form H-bonds as donors and acceptors and therefore, these peptides exhibit the potential to be used to treat HEV in future after their detailed clinical assessments.

## 4. Materials and Methods

### 4.1. Retrieval and Optimization of Ligand Molecules

The amino acid sequences of 500 antimicrobial peptides were retrieved from NCBI’s Entrez protein database (https://www.ncbi.nlm.nih.gov/protein; accessed on 12 November 2022) in FASTA format. The sequences were subjected to MEGA7 [34] software for manual alignment and to identify highly conserved or identical regions among all peptides. The sequences of the most frequently occurring peptides were used to devise tetrapeptides and their chemical structures were prepared using ACD ChemSketch [35] software in MOL format. The tetrapeptides were energy minimized and then converted into pdbqt format through Open Babel tool of PyRx. The prepared peptides were then used as ligands in molecular docking studies [36,37].

### 4.2. Retrieval and Refinement of the Receptor Protein

The 3D structure of the capsid protein of HEV was retrieved from Protein Data Bank (https://www.rcsb.org/; accessed on 12 November 2022) with PDB ID: 3HAG_A. No currently bound ligand was found in the downloaded 3D structure. If the protein has a bound ligand then the ligand has to be removed before molecular docking. The protein consisted of chain A only which was used in the docking experiments. The site finder tool of the Molecular operating environment [38] software was used for the prediction of the binding pocket with default parameters. The prediction of the active site is a critical and important step in molecular docking experiments. The receptor protein was prepared by removing water molecules, adding hydrogen atoms, 3D protonation, energy minimization with default parameters (i.e., Forcefield: MMFF94x, Constraints: Rigid water molecules, and Gradient: 0.1 RMS kcal/mol/A2).

### 4.3. Molecular Docking Analysis

PyRx software was used for molecular docking [39]. The prepared protein was then imported into PyRx and changed into pdbqt format and set as the receptor protein. A grid box was defined with dimensions (X: 5.1957, Y: 75.6891, Z: 55.3387) and with number of points (X: 65, Y: 106, Z: 87), spacing 0.9566 Å, and the value of exhaustiveness was set to its default (i.e., 8) to maximize the binding conformational investigation between protein and ligands. The docked poses with the lowest binding free energy and root mean square deviation (RMSD) were selected. The docking score between the receptor protein and ligand is used as a primary evaluation criterion to screen potential compounds and their putative target(s) [36,40]. Finally, the Discovery Studio [41] software was used for the visualization of interactions between the receptor protein and key active compounds.

### 4.4. Evaluation of Pharmacokinetics Parameters

The druggability assessment is an essential step for the evaluation of drug-like behavior of selected drug candidates. The druggability of top ligands was evaluated using SwissADME [42]. The pharmacokinetics properties were evaluated by an online server admetSAR [43]. The pharmacokinetics properties include absorption, distribution, metabolism, excretion, and toxicity (i.e., ADMET) based attributes of the selected compounds. Only those compounds could be further processed as leading drug candidates that fully accomplish the ADMET parameters.

### 4.5. Molecular Dynamics Simulation

The stability of the docked complexes was checked through molecular dynamics (MD) simulation. The top-ranked five complexes were used in MD simulation studies based on molecular interactions and visual examination. The Schrodinger’s Desmond Module was employed to perform MD simulations [44]. A water-soaked solvent solution was used to make predictions. The TIP3P water model is being investigated to resolve MD simulation-related problems. A box with periodic boundary conditions and a buffer distance of at least 10 Å from the outer surface off protein is used to create the orthorhombic simulation. To neutralize the system, an appropriate number of counter-ions were added and 0.15 M NaCl was added to maintain the simulation box’s isosmotic state. A predetermined equilibration process was followed before the simulation’s production run. The MD simulation was performed at a temperature of 310 K and a pressure of 1.013 bar. The simulation was run for 150 nanoseconds and 1000 frames were recorded in the trajectory. The simulation interaction diagram was used to analyze the MD simulation trajectory.

## 5. Conclusions

The acute hepatitis is caused by hepatitis E virus (HEV) in one-third of world’s population. The capsid protein of HEV is the core protein which is essential in viral replication and assembly. Until now, many drugs and vaccine candidates have been studied but many of them failed due to the variable nature of viral proteins. In the current study, we docked 100 tetrapeptides devised from conserved regions of 500 antimicrobial peptides as ligand molecules to the HEV capsid protein. The results of molecular docking, druggability, and molecular dynamics simulation studies have revealed the potential of these peptides as inhibitors of the capsid protein with no side or toxic effects. The chemical and biological behavior of plant-derived tetrapeptides make them potent drug candidates against the capsid protein as a receptor protein of the HEV genome.

## Figures and Tables

**Table 1:** Energy profiling of selected tetrapeptides as ligands of ORF2 (capsid protein) of HEV.

Sr. No.	Ligand	Structure	S-Score	Interacting Amino Acids *
1	GSTR	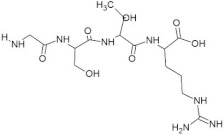	−10.1	Thr263, **Gln420**, Tyr443
2	TDGH	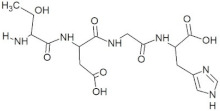	−8.5	Thr263, Ser264, Val364, **Arg399**, **Gln420**
3	LEEV	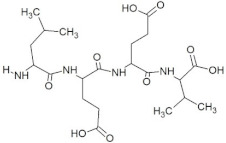	−8.4	Thr263, Ser264, Gly265, **Arg399**
4	WDDG	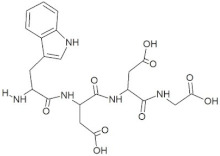	−8.2	**Arg366**, **Arg399**, **Gln420**
5	EGDE	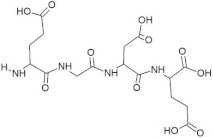	−8.0	Gly265, His354, Val364, **Arg399**, **Gln420**, **Asp444**
6	FTDG	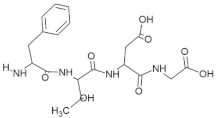	−7.8	Thr263, Val266, Val364, **Arg366**, **Arg399**, **Asp444**
7	EPST	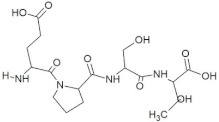	−7.5	Thr263, Ser264, Gly265, Val266, **Arg366**, **Asp444**, **Gln446**
8	SDAF	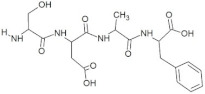	−6.5	**Val364**, **Arg366**, **Arg399**, Glu417
9	PRGS	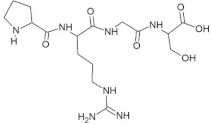	−5.7	Leu353, His354, **Arg366**, **Gln446**, Arg451, Thr453
10	NTFP	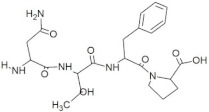	−5.6	Val364, **Arg399**, Glu417, **Gln420**, **Gln421**

* The active site residues reported previously have been shown in bold.

**Table 2:** Drug-like properties of selected antimicrobial peptides.

Peptides	Molecular Properties †						
	MW	HBD	HBA	nrotb	Log P	A	Violations
GSTR	419.44	10	8	17	−3.58	99.13	1
TDGH	428.40	8	8	12	−3.24	97.66	1
LEEV	488.54	7	7	16	−0.72	120.09	1
WDDG	491.46	8	7	16	−1.68	117.98	1
EGDE	448.39	8	8	15	−3.01	97.83	1
FTDG	438.44	7	7	12	−1.66	438.44	1
EPST	432.43	7	8	11	−3.16	103.31	1
SDAF	438.44	7	7	12	−1.69	105.51	1
PRGS	415.45	9	7	12	−2.96	104.67	1
NTFP	477.52	6	7	11	−1.68	122.96	1

MW: Molecular weight, HBD: Number of hydrogen bond donors; HBA: Number of hydrogen bond acceptors, nrotb: Number of rotatable bonds, log P: The logarithm of octanol/water partition coefficient, A: Molar refractivity.; † Molecular properties were calculated using SwissADME an online tool.

**Table 3:** ADMET related drug-like parameters of the best selected tetrapeptides.

	Peptides									
	GSTR	TDGH	LEEV	WDDG	EGDE	FTDG	EPST	SDAF	PRGS	NTFP
Absorption										
BBB	–	–	–	–	–	–	–	–	–	–
HIA	–	+	–	–	–	–	–	–	–	+
Caco-2 Permeability	Caco-2-	Caco-2-	Caco-2-	Caco-2-	Caco-2-	Caco-2-	Caco-2-	Caco-2-	Caco-2-	Caco-2-
PGS	Substrate	NS	NS	NS	NS	Substrate	Substrate	NS	Substrate	Substrate
PGI	NI	NI	NI	NI	NI	NI	NI	NI	NI	NI
ROCT	NI	NI	NI	NI	NI	NI	NI	NI	NI	NI
Metabolism										
CYP3A4 substrate	Substrate	Substrate	NS	Substrate	NS	NS	Substrate	NS	Substrate	Substrate
CYP2C9 substrate	NS	NS	Substrate	NS	NS	NS	NS	NS	NS	NS
CYP2D6 substrate	NS	NS	NS	NS	NS	NS	NS	NS	NS	NS
CYP3A4 inhibition	NI	NI	NI	NI	NI	NI	NI	NI	NI	NI
CYP2C9 inhibition	NI	NI	NI	NI	NI	NI	NI	NI	NI	NI
CYP2C19 inhibition	NI	NI	NI	NI	NI	NI	NI	NI	NI	NI
CYP2D6 inhibition	NI	NI	NI	NI	NI	NI	NI	NI	NI	NI
CYP1A2 inhibition	NI	NI	NI	NI	NI	NI	NI	NI	NI	NI
Toxicity										
AMES Toxicity	NAT	NAT	NAT	NAT	NAT	NAT	NAT	NAT	NAT	NAT
Carcinogens	NC	NC	NC	NC	NC	NC	NC	NC	NC	NC

BBB: Blood–Brain Barrier; HIA: Human Intestinal Absorption; PGS: P-glycoprotein substrate; PGI: P-glycoprotein inhibitor; ROCT: Renal Organic Cation Transporter; NS: Non-substrate; NI: Non-inhibitor; NAT: Non-Ames toxic; NC: Non-carcinogenic.

## Data Availability

Not applicable.

## Supplementary Materials

The following supporting information can be downloaded at: https://www.mdpi.com/article/10.3390/molecules28062675/s1, Figure S1: Interactions (a) and binding patterns (b) of WDDG peptide with ORF2 (capsid protein) of HEV as receptor; Figure S2: Interactions (a) and binding patterns (b) of EGDE peptide with ORF2 (capsid protein) of HEV as receptor; Figure S3: Interactions (a) and binding patterns (b) of FTDG peptide with ORF2 (capsid protein) of HEV as receptor; Figure S4: Interactions (a) and binding patterns (b) of EPST peptide with ORF2 (capsid protein) of HEV as receptor; Figure S5: Interactions (a) and binding patterns (b) of SDAF peptide with ORF2 (capsid protein) of HEV as receptor; Figure S6: Interactions (a) and binding patterns (b) of PRGS peptide with ORF2 (capsid protein) of HEV as receptor; Figure S7: Interactions (a) and binding patterns (b) of NTFP peptide with ORF2 (capsid protein) of HEV as receptor; Figure S8: Hydrogen bond interaction stability (consistency) of key residues of capsid protein with GSTR peptide; Figure S9: Hydrogen bond interaction stability (consistency) of key residues of capsid protein with TDGH peptide; Figure S10: Hydrogen bond interaction stability (consistency) of key residues of capsid protein with LEEV peptide; Figure S11: Hydrogen bond interaction stability (consistency) of key residues of capsid protein with WDDG peptide; Figure S12: Hydrogen bond interaction stability (consistency) of key residues of capsid protein with EGDE peptide.

## Sample Availability

Sequences and structures used in this study are available from the corresponding author upon request.
