# Supplementary material for: Identification of Plant Peptides as Novel Inhibitors of Orthohepevirus A (HEV) Capsid Protein by Virtual Screening
Source: Molecules. 2023 Mar 16;28(6):2675. doi: 10.3390/molecules28062675 (PMC10051542; doi:10.3390/molecules28062675)
Supplement: Supplementary file 1 [file molecules-28-02675-s001.zip › molecules-2175552-supplementary.pdf]

Supplementary Materials

Identification of Plant Peptides as Novel Inhibitors of Orthohepevirus A (HEV) Capsid Protein by Virtual Screening

Ghulam Mustafa [1,*], Hafiza Salaha Mahrosh [2], Syed Awais Attique [3,4], Rawaba Arif [5], Mohammad Abul Farah [6], Khalid Mashay Al-Anazi [6] and Sajad Ali [7]

[1] Department of Biochemistry, Government College University Faisalabad, Faisalabad 38000, Pakistan
[2] Department of Biochemistry, University of Agriculture Faisalabad, Faisalabad 38000, Pakistan
[3] School of Interdisciplinary Engineering & Science (SINES), National University of Sciences & Technology (NUST), Islamabad 44000, Pakistan
[4] Agency for Science, Technology and Research (A*STAR), Bioinformatics Institute, 30 Biopolis Street, Matrix, Singapore 138671, Singapore
[5] Department of Biochemistry, University of Jhang, Jhang 35200, Pakistan
[6] Department of Zoology, College of Science, King Saud University, P.O. Box 2455, Riyadh 11451, Saudi Arabia
[7] Department of Biotechnology, Yeungnam University, Gyeongsan 38541, Republic of Korea

* Correspondence: drghulammustafa@gcuf.edu.pk

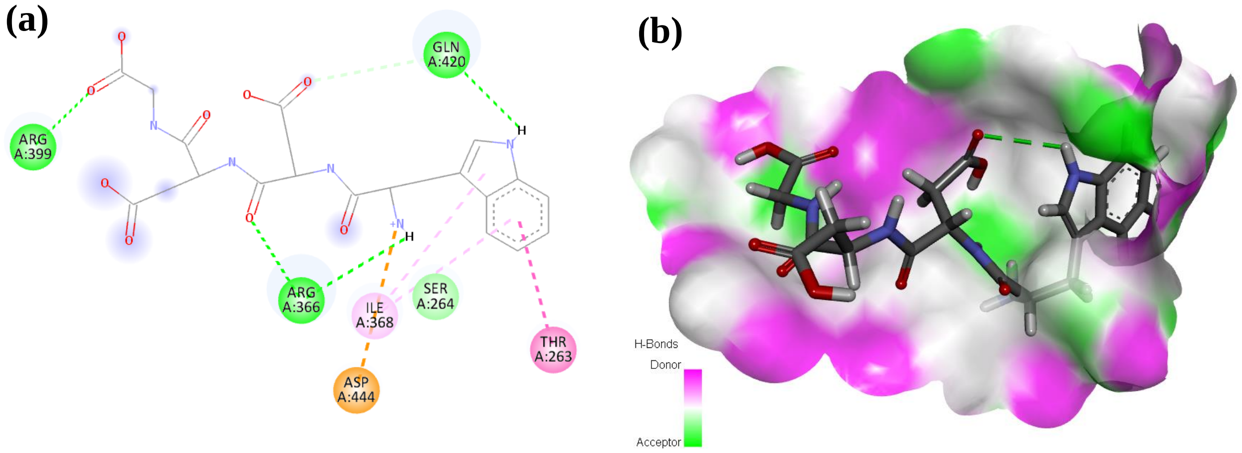

Figure S1. Interactions (a) and binding patterns (b) of WDDG peptide with ORF2 (capsid protein) of HEV as receptor.

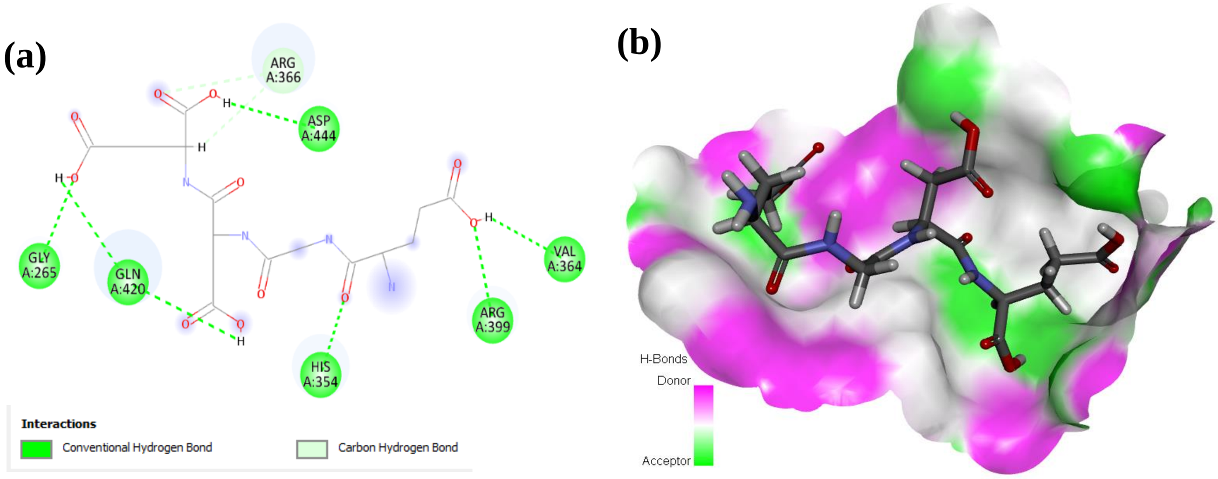

Figure S2. Interactions (a) and binding patterns (b) of EGDE peptide with ORF2 (capsid protein) of HEV as receptor.

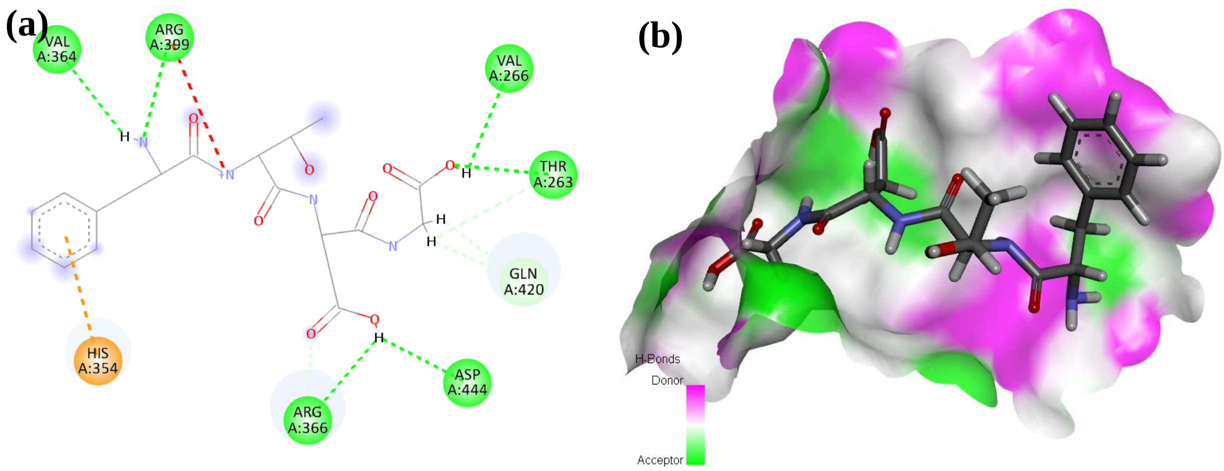

Figure S3. Interactions (a) and binding patterns (b) of FTDG peptide with ORF2 (capsid protein) of HEV as receptor.

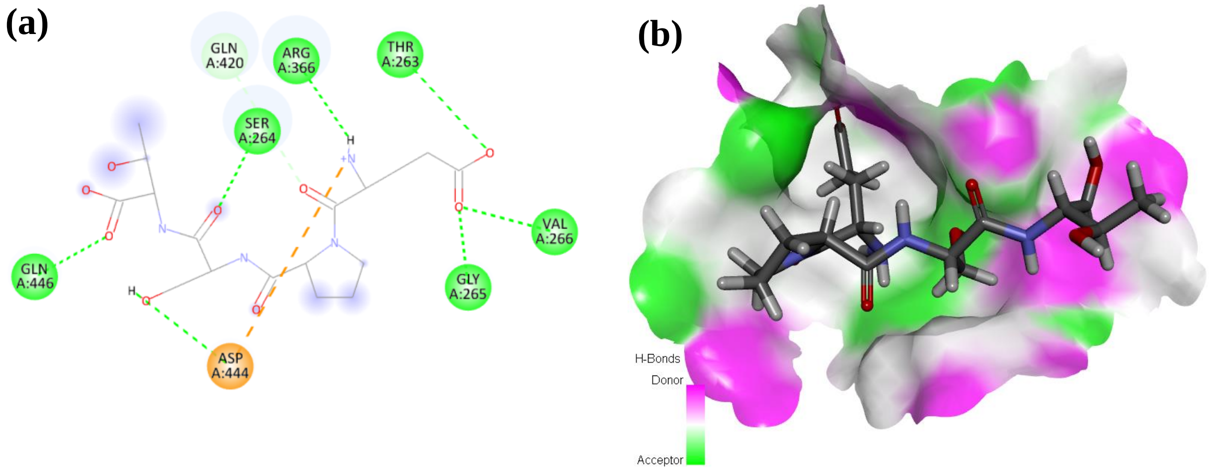

Figure S4. Interactions (a) and binding patterns (b) of EPST peptide with ORF2 (capsid protein) of HEV as receptor.

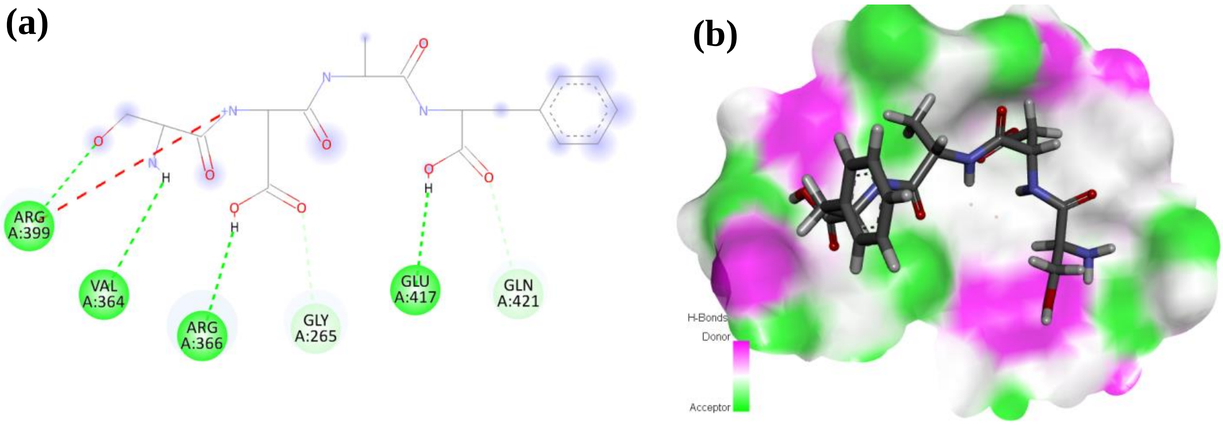

Figure S5. Interactions (a) and binding patterns (b) of SDAF peptide with ORF2 (capsid protein) of HEV as receptor.

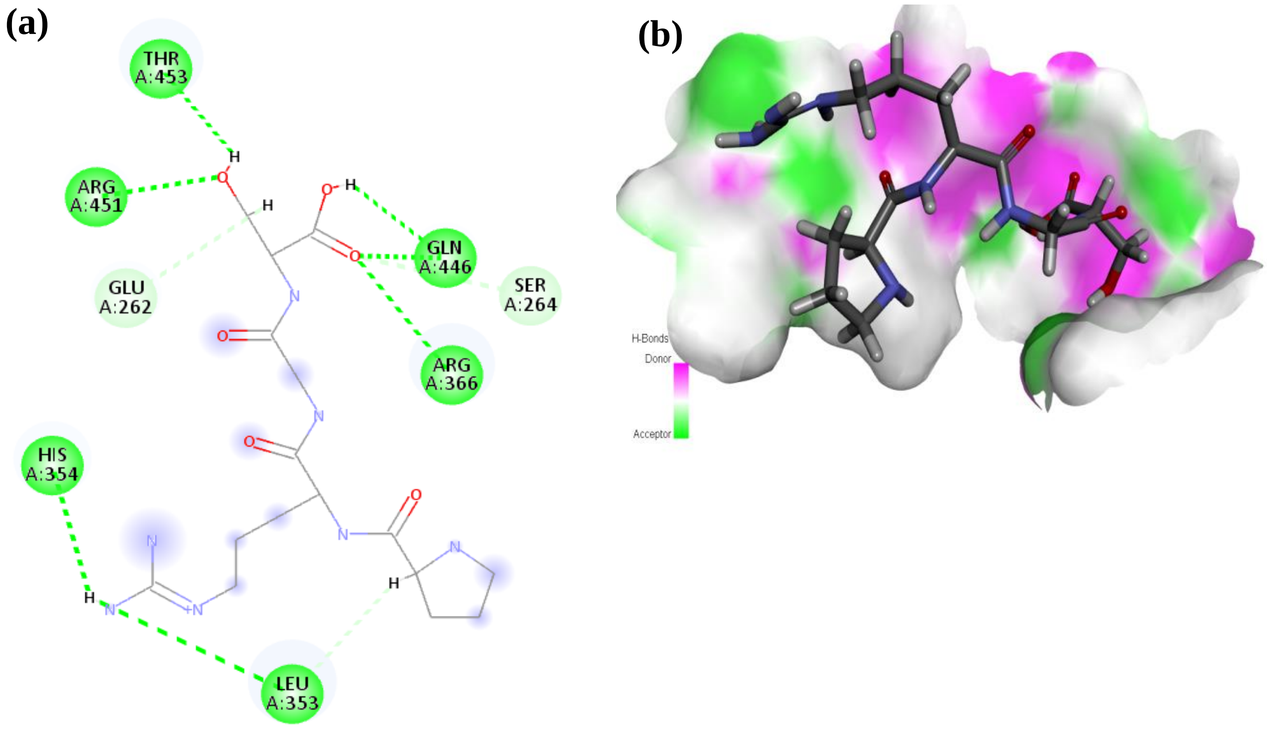

Figure S6. Interactions (a) and binding patterns (b) of PRGS peptide with ORF2 (capsid protein) of HEV as receptor.

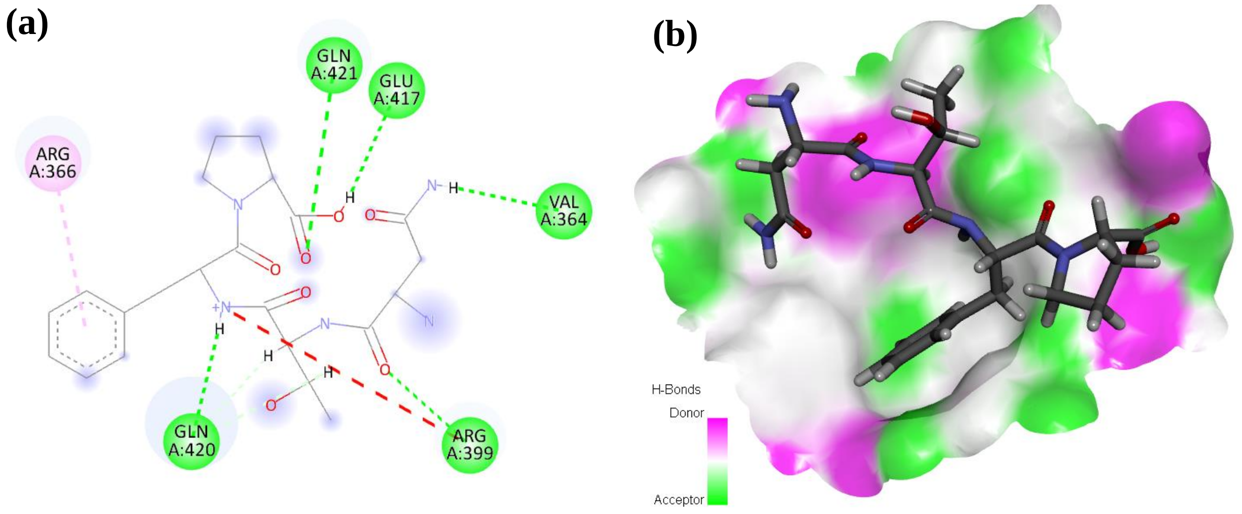

Figure S7. Interactions (a) and binding patterns (b) of NTFP peptide with ORF2 (capsid protein) of HEV as receptor.

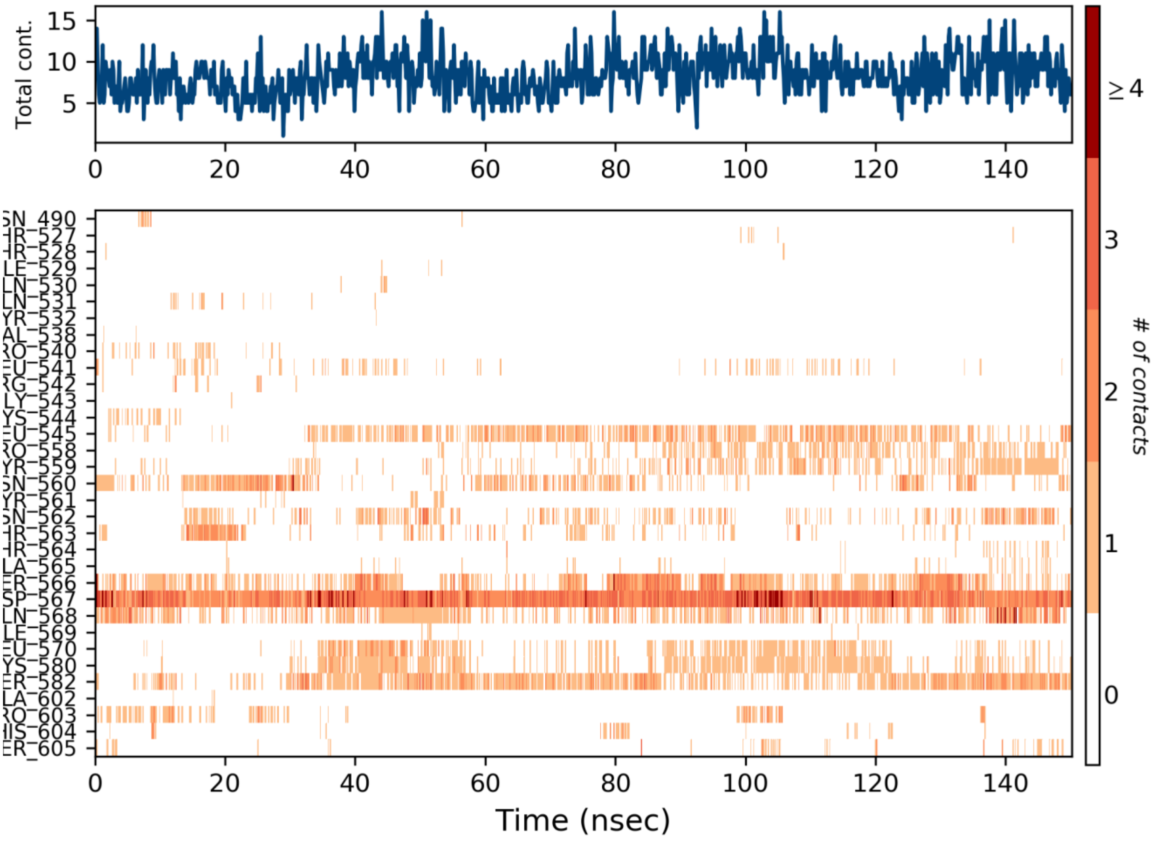

Figure S8. Hydrogen bond interaction stability (consistency) of key residues of capsid protein with GSTR peptide.

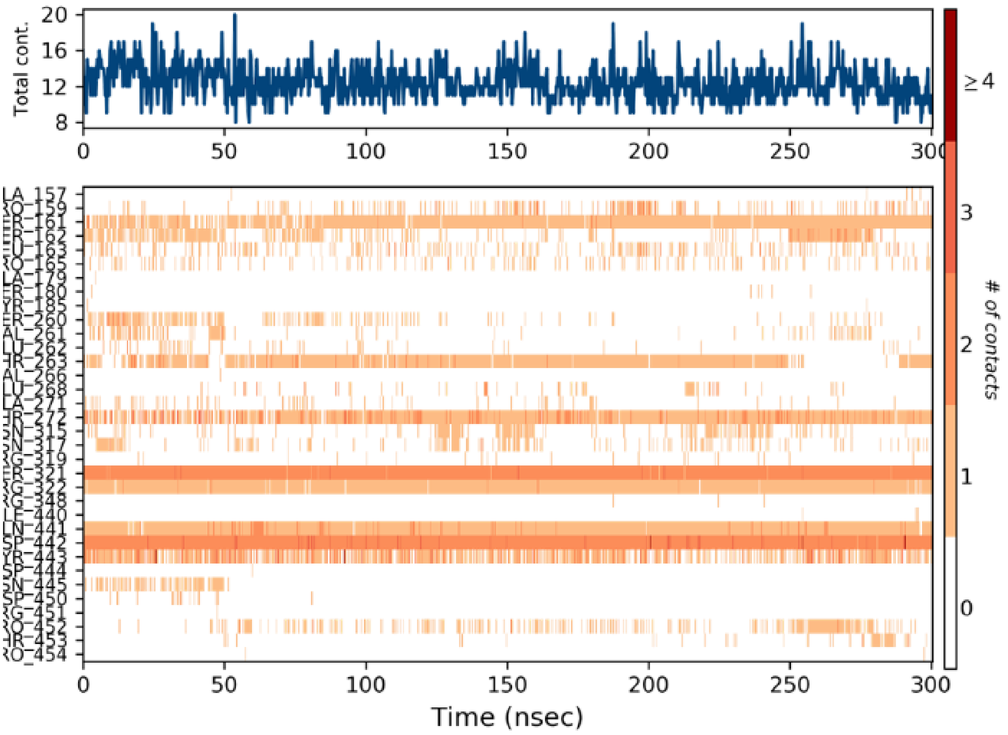

Figure S9. Hydrogen bond interaction stability (consistency) of key residues of capsid protein with TDGH peptide.

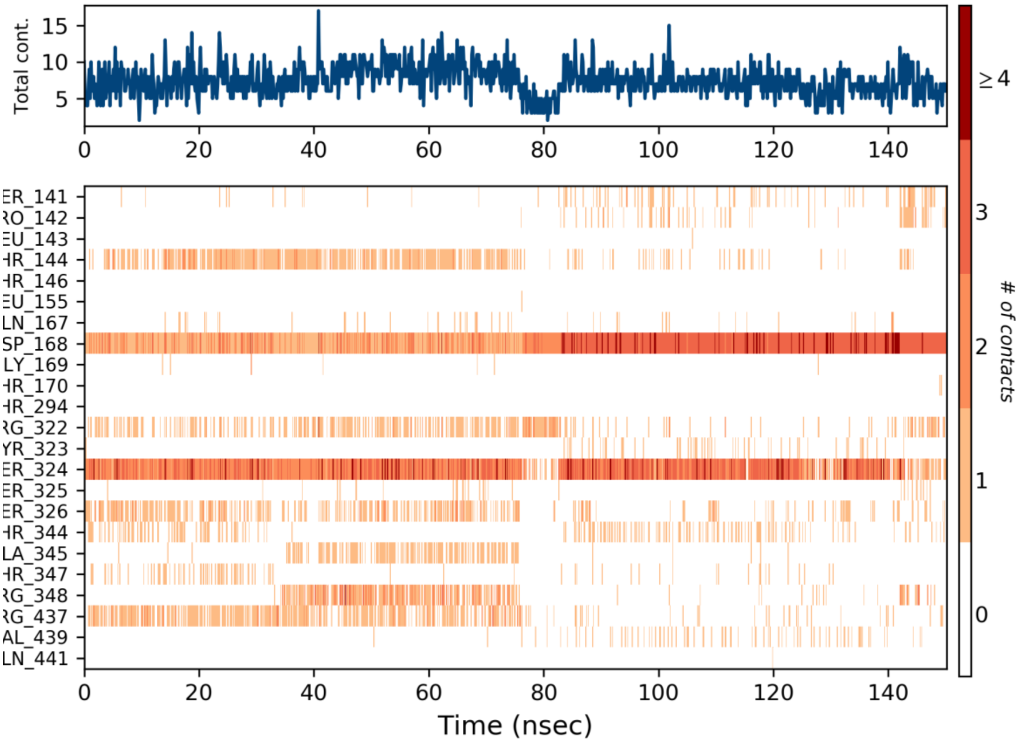

Figure S10. Hydrogen bond interaction stability (consistency) of key residues of capsid protein with LEEV peptide.

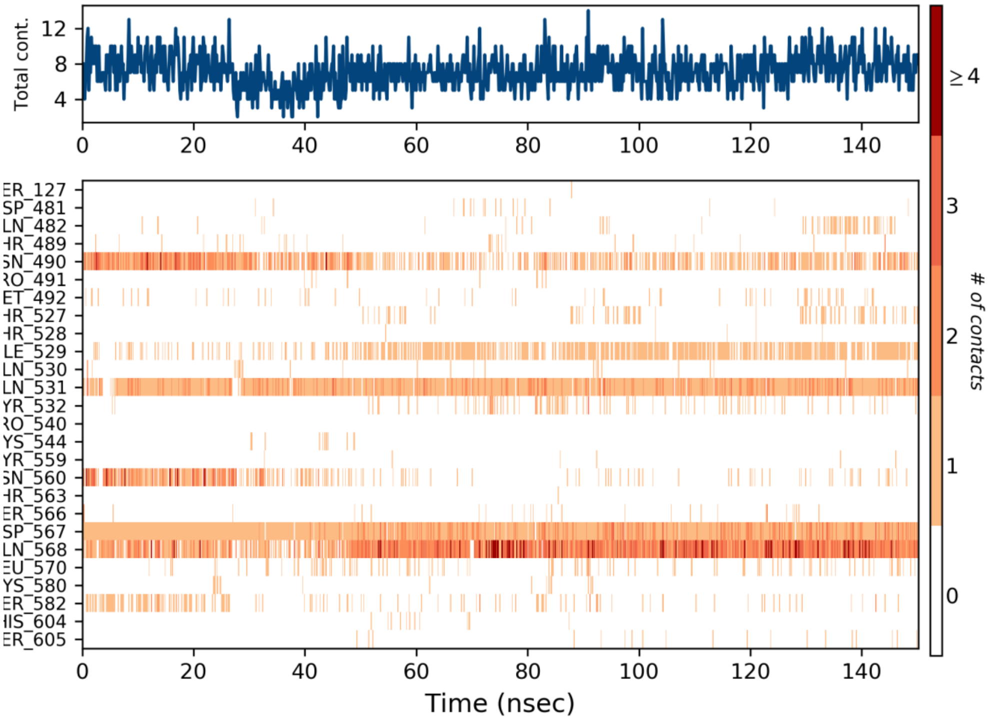

Figure S11. Hydrogen bond interaction stability (consistency) of key residues of capsid protein with WDDG peptide.

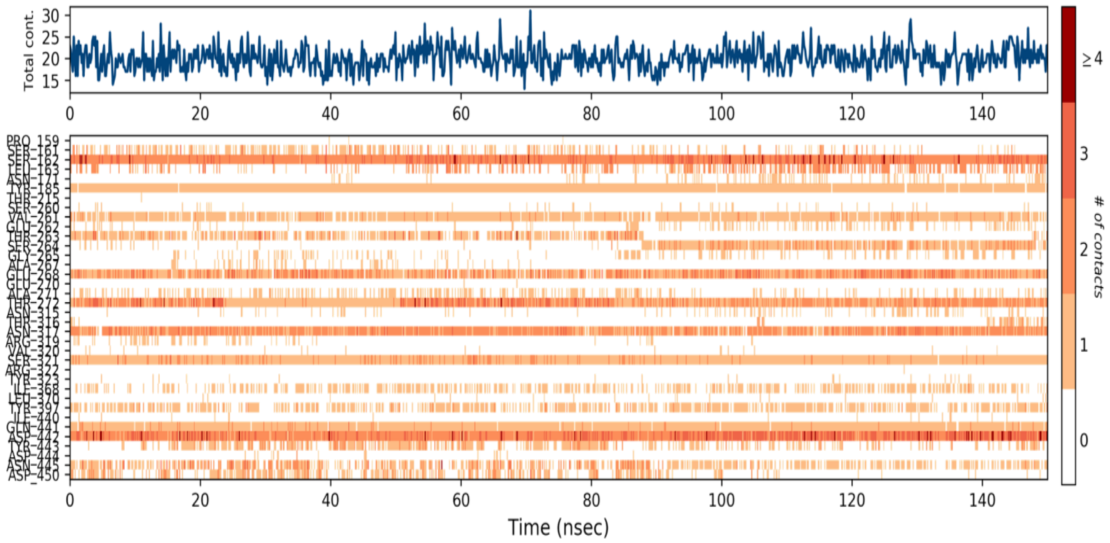

Figure S12. Hydrogen bond interaction stability (consistency) of key residues of capsid protein with EGDE peptide.
